# Physiological responses and energy cost of walking on the Gait Trainer with and without body weight support in subacute stroke patients

**DOI:** 10.1186/1743-0003-11-54

**Published:** 2014-04-10

**Authors:** Anna Sofia Delussu, Giovanni Morone, Marco Iosa, Maura Bragoni, Marco Traballesi, Stefano Paolucci

**Affiliations:** 1 Fondazione Santa Lucia, I.R.C.C.S, Via Ardeatina, 306, 00179 Rome, Italy

**Keywords:** Robotic training, Stroke rehabilitation, Energy cost of walking, Gait

## Abstract

**Background:**

Robotic-assisted walking after stroke provides intensive task-oriented training. But, despite the growing diffusion of robotic devices little information is available about cardiorespiratory and metabolic responses during electromechanically-assisted repetitive walking exercise. Aim of the study was to determine whether use of an end-effector gait training (GT) machine with body weight support (BWS) would affect physiological responses and energy cost of walking (ECW) in subacute post-stroke hemiplegic patients.

**Methods:**

Participants: six patients (patient group: PG) with hemiplegia due to stroke (age: 66 ± 15y; time since stroke: 8 ± 3 weeks; four men) and 6 healthy subjects as control group (CG: age, 76 ± 7y; six men).

Interventions: overground walking test (OWT) and GT-assisted walking with 0%, 30% and 50% BWS (GT-BWS0%, 30% and 50%). Main Outcome Measures: heart rate (HR), pulmonary ventilation, oxygen consumption, respiratory exchange ratio (RER) and ECW.

**Results:**

Intervention conditions significantly affected parameter values in steady state (HR: p = 0.005, V’E: p = 0.001, V'O_2_: p < 0.001) and the interaction condition per group affected ECW (p = 0.002). For PG, the most energy (V’O_2_ and ECW) demanding conditions were OWT and GT-BWS0%. On the contrary, for CG the least demanding condition was OWT. On the GT, increasing BWS produced a decrease in energy and cardiac demand in both groups.

**Conclusions:**

In PG, GT-BWS walking resulted in less cardiometabolic demand than overground walking. This suggests that GT-BWS walking training might be safer than overground walking training in subacute stroke patients.

## Background

Ambulation recovery is the main goal in rehabilitating stroke patients because of its role in improving autonomy and social participation [1]. As the rehabilitation of stroke patients is long and expensive, the first part of the process (during the in-patient phase) should be optimized. Research on novel rehabilitation approaches, such as programs to relearn how to walk, have aimed to improve functional outcomes and independence in the activities of daily living in a shorter recovery period [2]. The guiding principles of these novel approaches to walking recovery are task-oriented training [3,4] and frequency, intensity and duration of exercise [5,6]. Aerobic training has been shown to have some benefits in the functional recovery of stroke populations: it slows down the decline of physiological fitness reserves [7], improves walking capacity [8] and enhances selected cognitive domains responsible for better sensory motor control [9]. Other important benefits of aerobic training in stroke patients concern glucose tolerance and hyperinsulinemia [10] and endogenous fibrinolysis, which can reduce secondary myocardial and cerebral atherothrombotic risk [11]. As previously shown [12], this multi-systemic approach aims to improve both neurological and cardiological health outcomes.

One strategy used to increase the intensity of walking training (i.e. walking time and speed) is practice on the treadmill with partial body weight support. When this strategy was developed about 20 years ago to treat patients with brain damage, it showed little evidence of efficacy in stroke patients [13,14]. It was progressively developed using electromechanical steppers and robotic devices. The two most common commercial robotic [15] gait machines available for walking training in hemiplegic patients are the Gait Trainer (GT), which controls endpoint trajectories (GT II, Rehastim. Berlin) [16,17], and the Lokomat (Hocoma Medical Engineering Inc., Zurich), which integrates a robotic exoskeleton on a treadmill [18]. Despite the increasing use of these machines little is known about their utilization and patients’ physiological responses during robotic walking training [19,20].

In hemiplegic patients, walking energy expenditure varies with degree of weakness and spasticity. The walking oxygen demand of these patients is greater than that of healthy subjects matched for body size. Furthermore, the hemiplegic condition reduces gait efficiency and increases the energy cost of walking (ECW) up to twice that of able-bodied individuals [21].

Tailoring walking training to the cardiovascular and motor abilities of patients should increase the efficacy of intensive task-oriented walking training [22]. Electromechanical devices and robots, such as the GT, Lokomat or body-weight-support treadmill training, all have body weight support (BWS) that allows patients to safely perform intensive walking training for locomotor and cardiovascular systems [22].

The use of electromechanical devices has become customary in daily life. Recent studies have reported that these machines are adjunctive tools in rehabilitation strategy and that they have proven efficacious in some but not all stroke patients. Furthermore, greater benefits have been observed in severely affected patients and in those with lower levels of anxiety [23-25]. In these patients cardiovascular pathologies and metabolic and muscular deconditioning secondary to immobility during the acute poststroke phase) are commonly present. Thus, it is imperative to know how demanding intensive walking training is for the metabolism and the heart in the subacute stroke phase. David and co-workers investigated cardiopulmonary responses during machine-assisted and unassisted walking and reported that the machine-assisted condition was less demanding for patients than healthy subjects. Nevertheless, they failed to demonstrate the oxygen requirements in different BWS conditions [26].

To our knowledge, no studies have yet been published about ECW on the GT in patients with stroke. ECW measurement is a functional evaluation method used to evaluate physiological response to exercise; it is used in rehabilitation to determine the cardiopulmonary effects of disability on walking capability [27].

The aim of this study was to assess the following parameters in subacute phase stroke patients and healthy age- and body-size matched subjects: cardiac and metabolic responses, ECW while walking on the GT, with a BWS of 0%, 30% and 50% of the patient’s body mass, respectively, and overground walking.

## Methods

### Participants

Inclusion criteria for the patients were: first time ischemic stroke in subacute phase (i.e. in the past 3 months); a Functional Ambulation Category (FAC) scale score of 2- 3; ability to walk with supervision or minor help, also with aid/s, for 5 minutes; a disability score above 50 on the Barthel Index Scale; stroke severity between mild (Canadian Neurological Scale score: ≥ 8) and moderate (Canadian Neurological Scale score: 5-7); age 18-80 years. Exclusion criteria were: comorbidities or disabilities other than stroke affecting walking capability; mental deterioration (inability to understand or follow directions).

An age- and body size-matched healthy control group was also recruited. The study was approved by the independent Ethics Committee of the Fondazione Santa Lucia, I.R.C.C.S. (Rome, Italy). All participants were fully informed before they signed the consent form to take part in the study and all gave their permission to publish data and, if necessary, images.

### Walking tests

**Figure 1:**
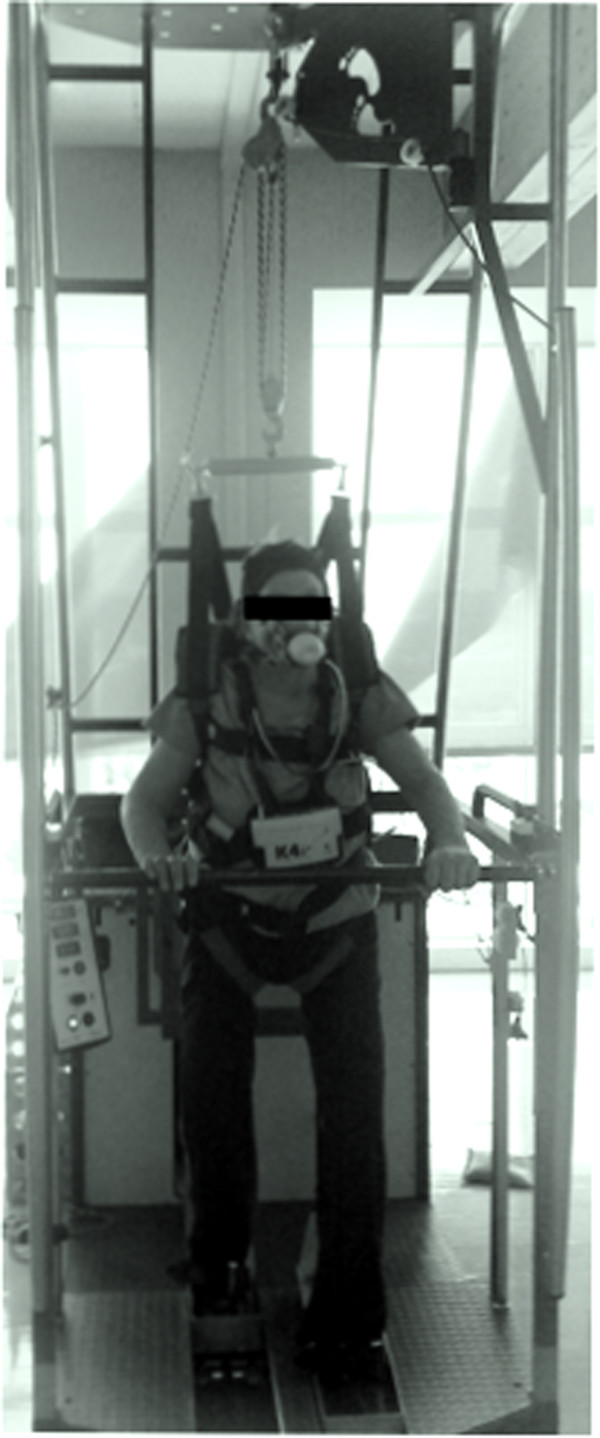
Patient on the Gait Trainer.

Each participant (i.e., both patients and controls) performed, in a randomized sequence on four consecutive days (always at the same time of the morning and with the same air temperature), an overground walking test (OWT) and three tests on the GT with BWS of 0%, 30% and 50% of their body mass (GT-BWS 0%, 30% and 50%). Figure 1 shows the set up for the walking test on the GT.

A few days before the walking tests, each participant performed at least two familiarization sessions with the GT to avoid learning effects.

In the OWT, all participants walked back and forth for at least 5 minutes on a 20 m long linear course at a comfortable self-selected walking speed (SSWS). If needed, patients were allowed to use their walking aid (e.g., cane or other). Patients were also supervised by a physiotherapist and/or physician. On the GT, walking speed was also self-selected by the participants. Speed was slowly but progressively increased until they chose their own SSWS (generally within the first minute of walking on the GT) from the speed choices available. The GT permits a maximal walking speed of 2 Km/h [22], which is slower than a healthy individual’s gait velocity. During the walking tests, patients and controls wore a breath-by-breath portable gas analyzer (K4b^2^, Cosmed, Italy) and a heart rate monitor (Polar Electro Oy, Finland). Before each test, the K4b^2^ was calibrated according to the manufacturer’s procedures. The following parameters were recorded during test performance: ventilation (V’E l/min), oxygen consumption (V’O_2_ ml/kg/min), carbon dioxide production (V’CO_2_ ml/kg/min), respiratory exchange ratio (RER, i.e. V’CO_2_/V’O_2_), heart rate (HR beats per minute- bpm) and walking speed (m/min). For the OWT, mean walking speed was calculated as the ratio of distance to time; thus, the walking speed obtained in the last 2 min of data collection was considered. Finally, as an index of the exercise intensity (EI) of walking, the percentage of predicted maximum heart rate (PMHR) was determined as follows: (SSHR/PMHR) * 100. PMHR was calculated according to Tanaka et al. [28] as follows:

208–0.7*age.

Both OWT and GT-BWS 0%, 30% and 50% lasted at least 5 minutes to allow participants to reach the steady state phase (SS) of the cardiac (HR) and metabolic parameters (V’E, V’O_2_, V’CO_2_ and RER). These data were collected before the tests began, i.e. during the rest condition and the test performance. The baseline data were computed as the mean value of the last 3 minutes of a10-minute resting condition recording, and the SS phase data were calculated as the mean value of the data collected in the last two minutes of data recording during each walking test.

Metabolic and cardiac data analysis was carried out offline. To determine ECW, only the SS phase data were considered. ECW was calculated as follows: SSV’O_2_ (ml/kg/min)/walking speed (m/min). Also, for each walking condition cardiac and metabolic data changes at SS were calculated as percentages of the resting values: [(SSHR–RestHR) * 100]/SSHR.

### Statistical analysis

All data are reported as means and standard deviations. A repeated measures ANOVA was carried out to assess differences among walking tests, including within (walking conditions: overground, BWS0%, BWS30%, BWS50%) and between (group: PG, CG) subjects factors. Walking conditions and group were considered as main factors in this analysis, thus the comparison among walking conditions was performed by including all subjects in the two groups; the group comparisons were performed by including all walking conditions. The level of significance for the ANOVA analysis was set at p ≤ 0.05. When ANOVA revealed statistically significant results, post-hoc comparisons were carried out with Bonferroni correction.

## Results

Six patients (patient group: PG) and six healthy subjects (control group: CG) were enrolled and completed all measurements. Their characteristics are reported in Table 1.

**Table 1:** Demographic and clinical features of patients (PG) and control (CG) groups

**Group**	**Participants**	**Sex**	**Age (years)**	**Body mass (kg)**	**Height (cm)**	**Body mass index (kg/m**^**2**^**)**	**FAC (score)**	**BI (score)**	**CNS (score)**	**Time since stroke (weeks)**
PG	P1	M	76	68	160	27	2	70	8.5	10
PG	P2	M	79	76	172	26	2	50	6.0	8
PG	P3	F	71	60	160	23	3	60	8.5	12
PG	P4	F	40	63	160	25	3	50	7.0	8
PG	P5	M	74	68	160	27	3	65	6.5	2
PG	P6	M	58	63	174	21	2	70	7.5	10
CG	C1	M	41	69	172	23	NA	NA	NA	NA
CG	C2	M	70	70	171	24	NA	NA	NA	NA
CG	C3	M	63	80	177	26	NA	NA	NA	NA
CG	C4	M	73	78	170	27	NA	NA	NA	NA
CG	C5	M	74	69	171	24	NA	NA	NA	NA
CG	C6	M	54	87	174	29	NA	NA	NA	NA
**PG**	Mean		66	66	164	24.6	3	60.8	7.3	8
	Standard deviation		15	6	7	2.2	1	9.2	1.0	3
**CG**	Mean		63	76	173	25.4	NA	NA	NA	NA
	Standard deviation		13	7	3	2.2	NA	NA	NA	NA
*t*-test	p-value		0.643	**0.039**	**0.020**	0.590	NA	NA	NA	NA

Legenda. *FAC* Functional Ambulation Classification, *BI* Barthel Index, *CNS* Canadian Neurological Scale, *NA* not assessed or not assessable. The last row reports the differences between PG and CG, in bold if statistically significant (p < 0.05).

Age was not statistically different between the two groups. Significant differences were found for stature and weight but not for the body mass index.

Table 2 reports cardiac and metabolic data of the four walking conditions of both groups as means and standard deviations. This table also reports results of analysis of variance for the within subject factor (i.e., walking condition), between subject factor (i.e., group) and their interaction. As expected, walking condition did not affect the values of the parameters collected during the rest phase, except for Rest RER, which was similarly affected by the walking condition in the two groups. Rest RER showed higher values in both groups in the overground walking condition with respect to all other conditions on the GT. This was especially true for healthy subjects when overground was compared with GT-BWS30%. Conversely, walking condition affected the values recorded during SS for RER, HR, EI, V’E, V’O_2_, but not ECW. SS RER resulted significantly different among walking conditions but not between groups; it was also significantly affected by the walking condition interaction per group. Post-hoc analyses revealed that RER in patients at the BWS0% was significantly higher than that evaluated overground (p = 0.007) and on the Gait Trainer at BWS50% (p = 0.012), but in healthy subjects RER was not significantly different among the four walking conditions. Between group post-hoc analyses showed no statistically significant differences between the two groups even for BWS0% (p = 0.045, no significant for Bonferroni correction). In any case, SS RER never reached 0.90 in either group. For HR, post-hoc analyses revealed a significant difference only for PG between OWT and GT-BWS30% (p = 0.0152); EI accounted for a submaximal effort [29] in both groups and in all walking conditions; it was significantly different among walking conditions, but not between groups or interaction. Post-hoc analyses of EI among walking conditions showed no statistically significant differences, even for GT-BWS30%, which was lower than the GT-BWS0% (p = 0.0412, no-significant for Bonferroni correction). For V’E, there were significant differences between OWT and GT-BWS30% and GT-BWS0% and GT-BWS30% for PG (p = 0.013 and p = 0.023, respectively) and OWT vs. GT-BWS50% for CG (p = 0.010). Concerning V’O_2_, PG showed significantly different values between OWT vs GT-BWS30% (p = 0.014) and GT-BWS50% (p = 0.019) and between GT-BWS0% vs GT-BWS30% (p = 0.009) and GT-BWS50% (p = 0.008). In the CG, a significant difference was observed only between OWT and GT-BWS50% (p = 0.0003). Although speed was significantly affected by walking condition, group and their interaction, post-hoc analyses revealed that this was due to a significant difference between groups only in the overground condition (p < 0.001). In fact, healthy subjects showed faster overground walking with respect to walking on the GT, whereas for patients no significant differences were detected among walking conditions.

**Table 2:** Cardiac and metabolic parameters in the four observed walking conditions

**Parameter**	**Group**	**Overground**	**GT-BWS 0%**	**GT-BWS 30%**	**GT-BWS 50%**	**ANOVA (p-value)**		
						**Walking conditions**	**Group**	**Interaction**
Rest RER	PG	0.77 ± 0.09	0.73 ± 0.03	0.73 ± 0.11	0.72 ± 0.07	**0.018**	0.635	0.944
	CG	0.79 ± 0.05	0.74 ± 0.07	0.74 ± 0.04^*^	0.75 ± 0.03			
SS RER	PG	0.78 ± 0.04^§^	0.86 ± 0.07	0.79 ± 0.07	0.76 ± 0.05^§^	**0.012**	0.379	**0.026**
	CG	0.76 ± 0.06	0.77 ± 0.06	0.77 ± 0.06	0.77 ± 0.08			
Rest HR (b/min)	PG	69 ± 8	80 ± 17	66 ± 8	73 ± 12	0.067	0.722	0.211
	CG	67 ± 8	71 ± 12	70 ± 11	72 ± 11			
SS HR (b/min)	PG	90 ± 12	103 ± 24	75 ± 5^*^	86 ± 18	**0.005**	0.951	0.366
	CG	91 ± 16	95 ± 20	85 ± 15	82 ± 9			
EI (%)	PG	58.7 ± 10	64.7 ± 16	49.2 ± 9*	53.7 ± 11	**0.005**	0.763	0.359
	CG	54 ± 11	58.4 ± 14	51.8 ± 11	50 ± 6			
Rest V’E (l/min)	PG	8 ± 2	8 ± 2	8 ± 2	8 ± 2	0.177	**0.011**	0.100
	CG	11 ± 2	11 ± 2	12 ± 2	13 ± 2			
SS V’E (l/min)	PG	21 ± 6	22 ± 7	14 ± 3^*§^	15 ± 5	**0.001**	**0.032**	0.688
	CG	26 ± 5	28 ± 9	23 ± 4	21 ± 5^*^			
Rest V’O_2_ (ml/kg/min)	PG	2.8 ± 0.9	3.2 ± 1.2	3 ± 1	3 ± 1.5	0.160	0.179	0.175
	CG	3.7 ± 0.7	3.4 ± 0.7	4 ± 1	4 ± 1.3			
SS V’O_2_ (ml/kg/min)	PG	11.5 ± 2.8	10.7 ± 2.9	9 ± 3.3^*§^	8 ± 3.2^*§^	**<0.001**	0.145	0.666
	CG	12.7 ± 2	13.8 ± 4.1	11 ± 1.8	10 ± 1.9^*^			
ECW (ml/kg/m)	PG	0.69 ± 0.4	0.42 ± 0.1	0.34 ± 0.1^§^	0.31 ± 0.1^§^	0.112	0.441	**0.002**
	CG	0.21 ± 0.0	0.52 ± 0.1^*^	0.43 ± 0.1^*^	0.36 ± 0.1^*^			
Speed (m/min)	PG	20.8 ± 8.4	25.5 ± 2.9	25.0 ± 2.9	25.1 ± 2.8	**<0.001**	**<0.001**	**<0.001**
	CG	60.0 ± 7.4^§^	26.1 ± 2.6^*^	25.6 ± 1.9^*^	27.0 ± 3.7^*^			

Legenda. BWS: Body-weight Support; PG: patient group; CG: control group; Rest RER: respiratory exchange ratio at rest; SS RER: respiratory exchange ratio at steady state; HR (beats/min): heart rate at rest; SS HR (beats/min): heart rate at steady state; EI: exercise intensity; Rest V’E (l/min): pulmonary ventilation at rest; SS V’E (l/min): pulmonary ventilation at steady state; Rest V’O_2_ (ml/kg/min): oxygen consumption at rest; SS V’O_2_ (ml/kg/min): oxygen consumption at steady state; ECW (ml/kg/m): energy cost of walking; speed (m/min): walking speed.

Bold characters indicate significant difference of post-hoc analysis: ^*^with respect to overground, ^§^with respect to GT-BWS0%.

**Figure 2:**
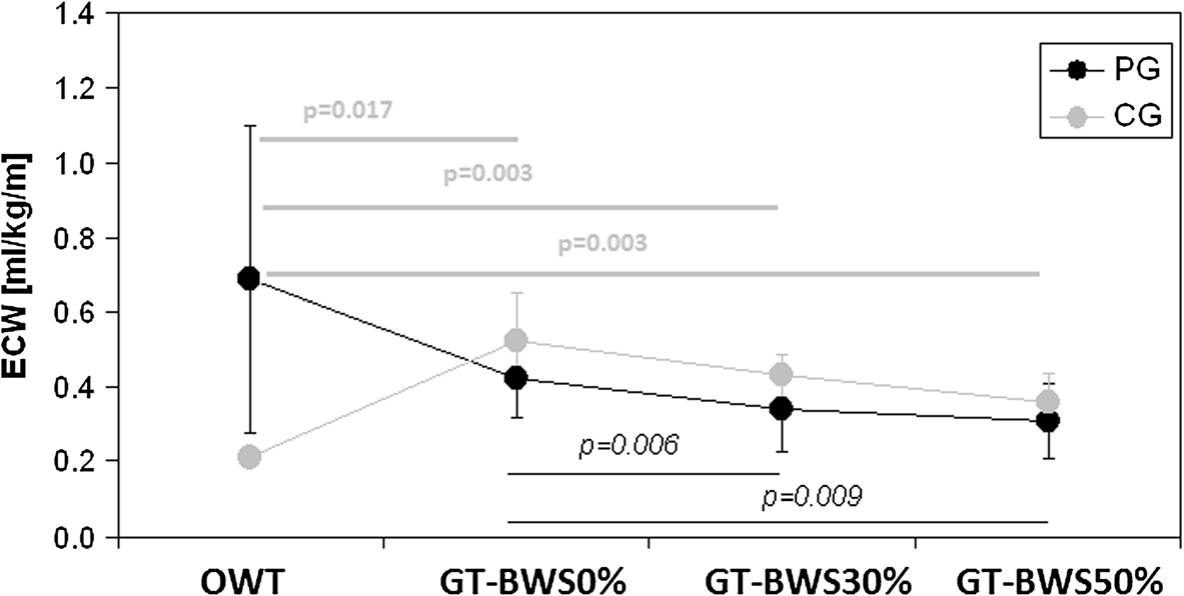
**Energy cost of walking results in the observed walking conditions in both groups.** Legenda. ECW: energy cost of walking measured in millilitres/kilogram/meter (ml/kg/m); OWT: overground walking test; GT-BWS 0%, 30%, 50%: walking tests on the Gait Trainer with 0%, 30% and 50% of subjects’ mass body weight support.

The factor group significantly affected V’E both at rest and SS, with lower values for PG. The walking condition per group interaction was also significant for ECW. The values and post-hoc analysis results for ECW are shown in Figure 2. A trend toward a progressive decrease in ECW was observed in PG from OWT to GT-BWS, which was proportional to the increase in BWS. The high variability recorded for patients on the OWT limited the statistically significant results in this condition; by contrast, on the GT significant differences were observed for GT-BWS0% vs GT-BWS30% (p = 0.006) and GT-BWS50% (p = 0.009). In control subjects, a similar trend was observed on the GT, but with the significantly lowest values of ECW recorded for walking overground (where, differently from patients, the variability between subjects was very low).

Results show that for PG the most energy (V’O_2_ and ECW) demanding conditions were OWT and GT-BWS0%. On the contrary, for CG the OWT was the least demanding task and on the GT the increase in BWS produced a decrease in energy and cardiac demand.

**Figure 3:**
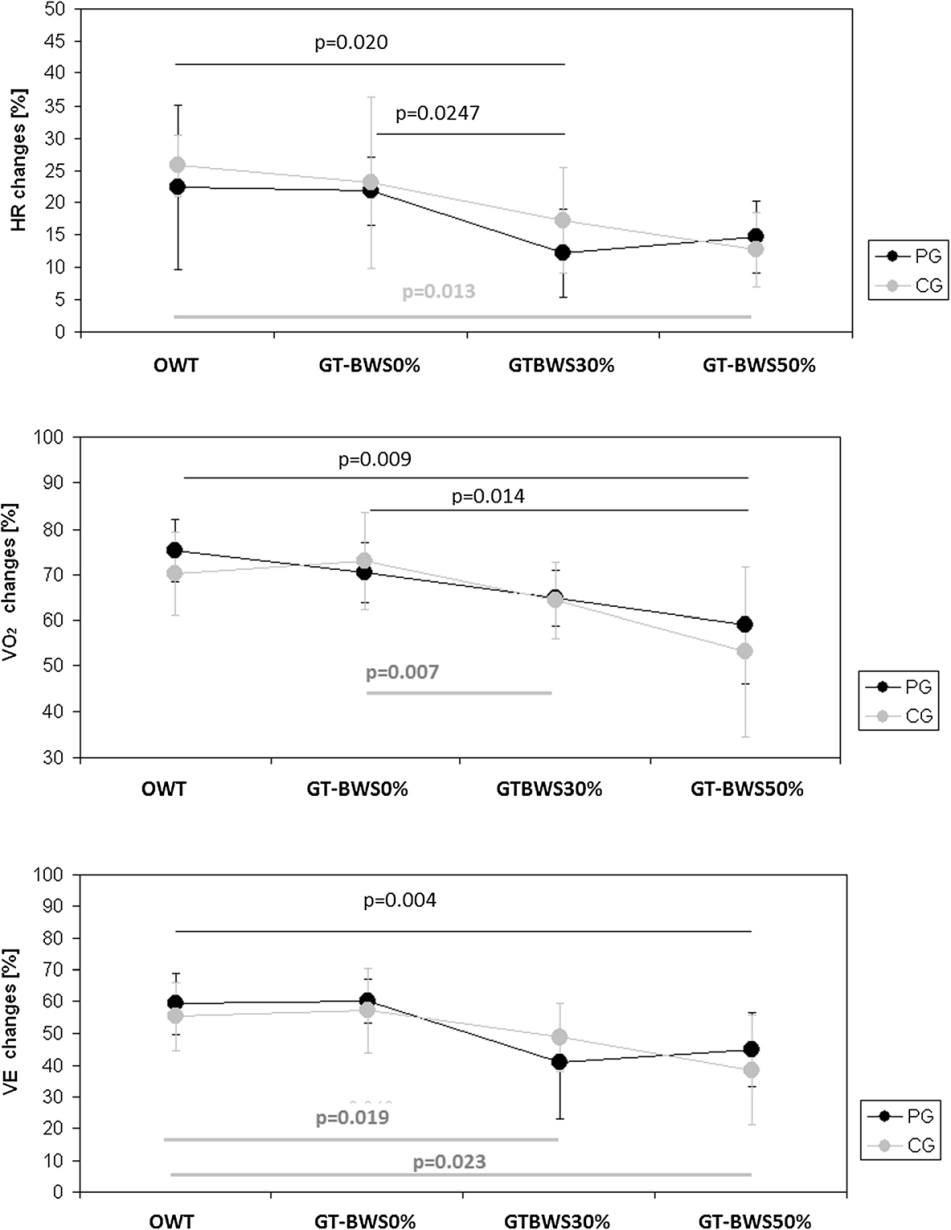
**Changes in cardiac and metabolic data at Steady State as percentages of resting values.** Legenda. HR: heart rate: V’O_2_: oxygen consumption; V’E: pulmonary ventilation; OWT: overground walking test; GT-BWS 0%, 30%, 50%: walking tests on the Gait Trainer with 0%, 30% and 50% subjects’ mass body weight support.

Figure 3 shows the changes in cardiac and metabolic data at SS considered as percentages of resting values.

Walking condition was statistically significant for HR (p = 0.004), V’E (p < 0.001) and V’O_2_ (p < 0.001). Note that there were no differences between groups (i.e., PG and CG) in any of the observed walking conditions.

As shown by the post-hoc analyses reported in Figure 3, in patients the GT-BWS30% was less demanding for both cardiac (HR changes) and metabolic (V’E changes) parameters (although the GT-BWS50% was less demanding than the GT-BWS0% for V’O_2_ changes). On the other hand, in the control group the OWT was the least demanding in terms of ECW, whereas on the GT the increase in BWS was paralleled by a decrease in cardiac and metabolic parameters, similar to what was observed in the patients.

## Discussion

The main results of the present study are the following: 1) in PG, when BWS increased walking on the GT led to reduced cardiac and metabolic requests; 2) in terms of ECW, walking overground was about three times more demanding for PG than CG; 3) in PG, walking on the GT without BWS was more demanding than walking overground and with GT-BWS30% and GT-BWS50%; 4), when expressed as percentage of resting values (Figure 3), cardiac and metabolic data showed a decrease in both groups with an increase in the percentage of BWS on the GT. Because of possible respiratory problems due to patients’ neurological conditions, which could have influenced the volume of gas exchanged, the latter result was particularly interesting.

We found that walking on the GT with 30 and 50% of BWS was less demanding than overground walking for the cardio-respiratory functions of patients with stroke. This finding is different from what was reported in previous studies using the same electromechanical device. In fact, no significant differences were found in oxygen consumption in the different BWS conditions [26]. The discrepancies between our results and those reported by David et al [26] could be due to differences in the way the walking tests were carried out. In that study, patients had to walk at a fixed speed in all walking tests and the healthy subjects had to walk at the same speed as the patients; in our study, speed was always self-selected by both groups of participants. Conversely, our results are in accordance with those found using another device for robotic-assisted gait training in stroke patients [30].

Our first result provides strong support for the use of electromechanically-assisted intensive walking training in cerebrovascular patients who often have cardiovascular diseases. This training can also have aerobic benefits (e.g., associated with glucose tolerance, hyperinsulinemia and endogenous fibrinolysis [10,11]).

The main difference between healthy subjects and patients was found for walking overground in terms of ECW (statistically significant condition per group interaction). The group main factor affected RER and V’E at rest and at steady state. Concerning Rest RER, in the overground condition both groups showed the highest value, which reached statistical significance in CG when compared with GT-BWS30%: SS RER showed no differences between groups and within CG, whereas in PG it resulted significantly higher than in the other walking conditions in the GT-BWS0%. The explanation of this result could be that the effort, even if submaximal (the RER never reached the value 0.90 [31]), was greater on the GT-BWS0% than the other walking condition as shown by SSHR, SSV’O_2_, SSV’E and ECW. On the basis of the RER data, it emerged that both groups used the same energetic substrate at rest and during all walking condition performances. PG showed a different energetic substrate than CG only on the GT-BWS0%; however, it was not statistically significant.

As already showed by the SS RER data, EI also accounted for a submaximal effort in both groups in all observed walking conditions; in fact, EI never reached 70% [28]. Concerning ventilation, PG showed lower ventilation values than CG at both rest and steady state. This could be due to the reduced and asymmetric strength of the patients’ respiratory muscles secondary to the post-stroke condition.

Finally, condition affected the values of HR, V’E and V’O_2_ at SS. Post-hoc analyses showed that these differences occurred among all conditions except GT-BWS30% and GT-BWS50%.

Likely, there was no statistically significant difference between the GT-BWS30% and GT-BWS50% conditions because during training GT-BWS30% was sufficient to compensate the patients’ impairment. As affirmed in recent studies, tailoring machine conditions to the patient’s ability is fundamental to optimize robotic-assisted therapy [22,23].

Our second result is in line with reports in the literature: a greater increase in oxygen consumption was already observed when patients walked overground [21] rather than on the treadmill with BWS [32]. High variability was also observed among patients during overground walking. In fact, it was greater than that observed on the GT-BWS0%, probably because the GT imposes standardized gait patterns.

Our third result was the opposite effect in healthy subjects: overground walking was less demanding than walking on the GT. This could have been because the GT imposes non-natural trajectories during walking-like training [22,24], which force subjects to activate non-natural sensorimotor walking patterns. This augments energy expenditure with respect to natural walking and, thus, exploits the mechanisms of energy recovery. Another reason for this effect could have been the slow walking speed imposed by the GT in healthy subjects, which also implies reduced walking energy efficiency (i.e. an increase in ECW). Conversely, for stroke patients balance control may benefit from harnessed trunk and handrail support (as on the GT) and reduce ECW with respect to overground walking [33]. This is in accordance with Christman and coworkers’ [34] study in which using a handrail was found to reduce heart rate and V˙O_2_ during treadmill walking.

The fourth result was that cardiac and metabolic data, when expressed as percentage of resting values, had a comparable trend (a decrease in cardiometabolic response paralleling the BWS increase) in both groups in GT WTs. Even if for CG this trend produced a reduction in ECW with the increase in BWS, on the GT the ECW was always higher than that registered overground. This was due to the limited speed allowed on the GT, which caused a significant reduction in walking speed for CG on the GT with respect to walking overground. On the GT, the walking speeds of CG were very close to those of PG. This was responsible for lowering the gait efficiency of CG on the GT.

It is crucial for therapists and physicians to obtain physiological response information about patients during robotic walking training because it can be used to create and administer rehabilitation programs. Physiological parameters have also been used to quantify the effects of different levels of mental engagement during walking training through recordings of ECG, breathing and skin temperature [35]. Online recording of psychological state using physiological markers of patients during robotic training can potentially improve rehabilitation outcomes, especially if this information is used to adjust the stimulus of the performed task [35].

The main limit of our study was small sample size. Nevertheless, many instrumental measures were taken, which highlight significant differences between groups and among conditions. Furthermore, because of the importance of tailoring training to individuals and being able to compare results with the findings of previous studies, we also tested participants at their self-selected walking speed [26]. Future studies should also investigate the effects of different speeds on cardio-respiratory parameters and measure actual BWS, which might be different from selected BWS [22].

## Conclusions

Our data suggest that robotic gait training, performed with body weight support, is a safe way for non-autonomous, ambulatory patients with subacute stroke (such as our patients) to engage in intensive walking training because the cardio-respiratory demand is lower than walking overground.

## Abbreviations

BWS: Body weight support (%); CG: Control group; ECW: Energy cost of walking (ml/kg/m); EI: Exercise intensity (%); GT: Gait trainer; GT-BWS0%, 30% and 50%: walking test on the GT with 0%, 30% and 50% BWS; HR: Heart Rate (beats per minute, bpm); OWT: Overground walking test; PG: Patient group; Rest HR: Heart rate at rest (beats per minute, bpm); Rest RER: Respiratory exchange ratio at rest; Rest V’E: Pulmonary ventilation at rest (l/min); Rest V’O2 (ml/kg/min): Oxygen consumption at rest (ml/kg/min); SS HR: Heart rate at steady state (beats per minute, bpm); SS RER: Respiratory exchange ratio at steady state; SS V’E: Pulmonary ventilation at steady state (l/min); SS V’O2: Oxygen consumption at steady state (ml/kg/min).

## Competing interests

The authors declare that they have no competing interests.

## Authors’ contributions

ASD designed the study, gathered data, contributed to data analysis and interpretation and drafted the manuscript; GM contributed to designing the study, acquiring and interpreting the data and drafting the manuscript; MI carried out the statistical analysis and contributed to interpreting the data and revising the manuscript; MB contributed to designing the study and revising the manuscript; MT contributed to designing the study and revising the manuscript; SP contributed to interpreting the data and revising the manuscript. All authors read and approved the final manuscript.
